# Supplementary figures and images for: Investigation of biometabolites and novel antimicrobial peptides derived from promising source Cordyceps militaris and effect of non-small cell lung cancer genes computationally
Source: PLoS One. 2025 Jan 23;20(1):e0310103. doi: 10.1371/journal.pone.0310103 (PMC11756765; doi:10.1371/journal.pone.0310103)

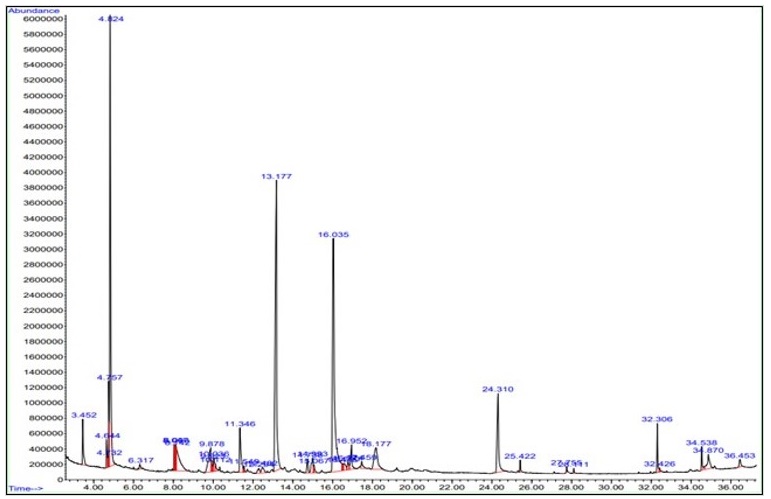

Supplement: S1 Fig — (JPG) [file pone.0310103.s001.jpg]

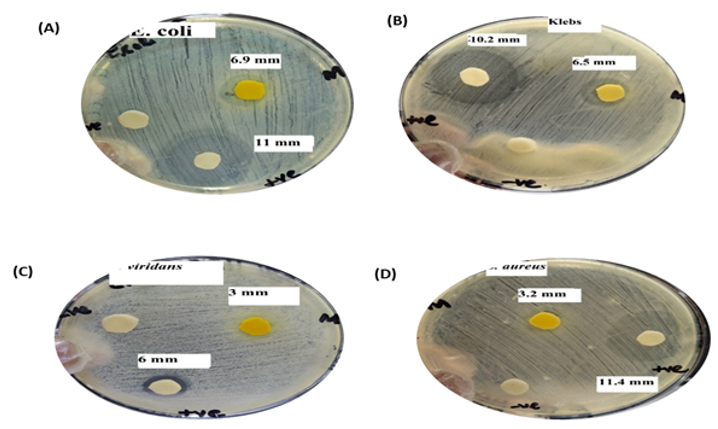

Supplement: S2 Fig — (A) E. coli (B) K. pneumoniae (C) S. viridans (D) S. aureus. (PNG) [file pone.0310103.s002.png]
