# Supplementary material for: Investigation of biometabolites and novel antimicrobial peptides derived from promising source Cordyceps militaris and effect of non-small cell lung cancer genes computationally
Source: PLoS One. 2025 Jan 23;20(1):e0310103. doi: 10.1371/journal.pone.0310103 (PMC11756765; doi:10.1371/journal.pone.0310103)
Supplement: S1 Table — (PDF) [file pone.0310103.s003.pdf]

S1 Table. GC-MS analysis of methanol extract of *C. militaris*.

Sr. no.	Bioactive Compounds	Mol. Wt. (g/mol)	Chemical formula	Retention time (min)	% Area
1	Furfural	96.08	$C_5H_4O_2$	4.644	1.11
2	3-Amino-2-methylbutanoic acid	117.15	$C_5H_{11}N0_2$	4.732	0.23
3	Pentanal	86.13	$C_5H_{10}O$	6.317	0.27
4	Cyclopentanone	84.12	C_5H_8O	8.047	0.66
5	6-Azacytosine	244.20	$C_8H_{12}N_4O_5$	8.090	1.47
6	2 (5H)-Furanone	84.07	$C_4H_4O_2$	8.142	3.70
7	2- Furancarboxylic acid	112.08	$C_5H_4O_3$	9.878	2.65
8	4,5-Diamino-6-hydroxypyrimidine	126.12	$C_4H_6N_4O$	9.923	0.50
9	Methyl furan-3-carboxylate	126.11	$C_6H_6O_3$	10.036	0.76
10	Methyl furan-3-carboxylate	126.11	$C_6H_6O_3$	10.112	0.42
11	4H-Pyran-4-one, 2,3-dihydro-3,5-dihydoxy-6- methyl-	144.12	$C_6H_8O_4$	11.346	2.91
12	Cyclobutanol	72.11	C_4H_8O	11.549	0.25
13	Alanylglycine, TMS	146.14	$C_5H_{10}N_2O_3$	12.294	0.42
14	Pentanal	86.13	$C_5H_{10}O$	12.482	0.65
15	5-Hydroxymethylfurfural	126.11	$C_6H_6O_3$	13.177	19.61
16	Guanidine, N,N-dimethyl-	87.12	$C_3H_9N_3$	14.738	0.78
17	Methyl[(3-methyl-1,2,4-oxadiazol-5-yl)methyl]amine	127.14	$C_5H_9N_3O$	14.993	1.23
18	Phenethylamine, p,.alpha.-dimethyl-	205.34	$C_{14}H_{23}N$	15.067	0.38
19	1,2,3-Benzenetriol	126.11	$C_6H_6O_3$	16.035	21.77
20	1,2,3-Benzenetriol	126.11	$C_6H_6O_3$	16.475	0.32
21	1,2,3-Benzenetriol	126.11	$C_6H_6O_3$	16.590	0.75
22	1,2,3-Benzenetriol	126.11	$C_6H_6O_3$	16.774	0.49
23	Trans-Cinnamic acid	148.16	$C_9H_8O_2$	16.952	1.55
24	Propanenitrile, 3-(methylamino)-	84.12	$C_4H_8N_2$	17.459	0.50
25	1,6-Anhydro-beta-D-glucopyranose	162.14	$C_6H_{10}O_5$	18.177	3.44
26	Benzoic acid, 3,4,5-trihydroxy-, methyl ester	184.15	$C_8H_8O_5$	24.310	5.45
27	n-Hexadecanoic acid	256.42	$C_{16}H_{32}O_2$	25.422	0.38
28	1,2-Benzenediol, 4-(2-amino-1-hydroxypropyl)-	169.18	$C_8H_{11}NO_3$	27.755	0.11
29	Propanamide	73.09	C_3H_7NO	28.111	0.16
30	2-Octen-4-one, 2-(methylamino)-	155.24	$C_9H_{17}NO$	32.306	1.62
31	Methyl[4-(methylamino)butyl]amine	116.20	$C_6H_{16}N_2$	32.426	0.17
32	Octadecanoic acid, 2,3-dihydroxypropyl ester	358.6	$C_{21}H_{42}O_4$	34.538	0.79
33	1,4-Benzenedicarboxylic acid, bis(2-ethylhexyl) ester	390.6	$C_{24}H_{38}0_4$	34.870	1.52
34	Indolizine, 2-(4-methylphenyl)-	207.27	$C_{15}H_{13}N$	36.453	0.51
