# Supplementary material for: Investigation of biometabolites and novel antimicrobial peptides derived from promising source Cordyceps militaris and effect of non-small cell lung cancer genes computationally
Source: PLoS One. 2025 Jan 23;20(1):e0310103. doi: 10.1371/journal.pone.0310103 (PMC11756765; doi:10.1371/journal.pone.0310103)
Supplement: S2 Table — (PDF) [file pone.0310103.s004.pdf]

S2 Table. LC-MS analysis of methanol extract of *C. militaris*.

Peak no.	Bioactive Compounds	Monoisotopic mass m/z	M + 1	Chemical formula	RT (min)	Product ions
1	Malonic acid	104.01	105	$C_3H_4O_4$	0.094	104, 156, 229, 318
2	1,2,3-Trihydroxybenzene	126.03	127	$C_6H_6O_3$	2.508	104, 126, 176, 248
3	Malonic acid	104.01	105	$C_3H_4O_4$	2.776	104
4	Hydroxypyruvic acid	104.01	105	$C_3H_4O_4$	2.901	104, 257
5	Dihydro-3-hydroxy-4,4-dimethyl- 2(3H)-Furanone	130.06	131	$C_6H_{10}O_3$	3.169	104, 130, 204
6	2-Aceto-2-hydroxy-butanoate	145.05	146	$C_6H_9O_4$	3.428	104, 236, 309, 364
7	5-Ethoxy-4,5-dihydro-2(3H)furanone	130.06	131	$C_6H_{10}O_3$	6.173	104, 130, 174, 240, 321
8	Caftaric acid	312.04	313	$C_{13}H_{12}O_9$	10.026	104, 130, 194, 268, 312, 376, 491
9	(E)-1-O-Cinnamoyl-beta-D-glucose	310.10	311	$C_{15}H_{18}O_7$	11.001	104, 144, 194, 246, 312, 376
10	4-Ethoxy-4-oxobutanoic acid	146.05	147	$C_6H_{10}O_4$	11.756	104, 146, 229, 291, 347
11	Oxoadipic acid	160.03	161	$C_6H_8O_5$	13.061	114, 160, 319
12	2-Methyltetrahydrofuran-3-one	100.05	101	$C_5H_8O_2$	13.265	100, 160,319
13	3,3’,4’,5,5’,8-Hexahydroflavone	318.03	319	$C_{15}H_{10}O_8$	13.839	100,160, 319
14	2-Hydroxyenterodiol	318.14	319	$C_{18}H_{22}O_5$	13.902	104, 160, 319
15	Trans-Aconitic acid	174.01	175	$C_6H_6O_6$	14.610	114, 174, 273, 347
16	5,8-Dihydroxy-3,3’,4’,7-tetramethoxyflavone	347.10	348	$C_{19}H_{18}O_8$	14.846	104, 174, 347
17	Aconitic acid	174.01	175	$C_6H_6O_6$	15.129	104, 174, 230, 347, 403
18	3-Methoxy-4-hydroxy-5-all-trans-heptaprenylbenzoate	643.47	644	$C_{43}H_{63}O_4$	20.256	581, 643,713,771, 849, 935
19	Cyanidin 3-sambubioside	581.15	582	$C_{26}H_{29}O_{15}$	20.507	581,643, 713, 771, 849, 935
20	Ampeloside Bs1	934.47	935	$C_{45}H_{74}O_{20}$	21.766	805, 895, 935, 968
