# Supplementary material for: Investigation of biometabolites and novel antimicrobial peptides derived from promising source Cordyceps militaris and effect of non-small cell lung cancer genes computationally
Source: PLoS One. 2025 Jan 23;20(1):e0310103. doi: 10.1371/journal.pone.0310103 (PMC11756765; doi:10.1371/journal.pone.0310103)
Supplement: S3 Table — (PDF) [file pone.0310103.s005.pdf]

S3 Table. Binding pockets position and their scores of Aldose Reductase protein by DeepSite.

Site No.	Scores	Centres
1	0.99436	[17.360000610351562, -12.920000076293945, -5.900000095367432]
2	0.99692	[7.360000133514404, 3.0799999237060547, 0.10000000149011612]
3	0.93079	[5.360000133514404, 21.079999923706055, 26.100000381469727]
4	0.84844	[31.360000610351562, 5.079999923706055, -7.900000095367432]
5	0.82873	[21.360000610351562, 11.079999923706055, 26.100000381469727]
