# Supplementary material for: Investigation of biometabolites and novel antimicrobial peptides derived from promising source Cordyceps militaris and effect of non-small cell lung cancer genes computationally
Source: PLoS One. 2025 Jan 23;20(1):e0310103. doi: 10.1371/journal.pone.0310103 (PMC11756765; doi:10.1371/journal.pone.0310103)
Supplement: S4 Table — (PDF) [file pone.0310103.s006.pdf]

S4 Table. Molecular Docking of Bioactive Compound of GC-MS analysis with RET, PIK3CA and TCTN3 Gene.

**Compounds**	**PUBCHEM I.D.**	**RET Affinity (kcal/mol)**	**PIK3CA Affinity (kcal/mol)**	**TCTN3 Affinity (kcal/mol)**
Indolizine, 2-(4-methylphenyl)-	346948	-8.0	-7.5	-6.9
6-Azacytosine	65114	-6.1	-6.8	-7.4
Benzoic acid, 3,4,5-trihydroxy-, methyl ester	7428	-6.0	-6.5	-6.2
1,2-Benzenediol, 4-(2-amino-1-hydroxypropyl)-	951	-6.0	-6.1	-6.3
1,2,3-Benzenetriol	1057	-5.3	-5.8	-6.0
Trans-Cinnamic	444539	-6.2	-5.5	-6.1
n-Hexadecanoic	985	-4.7	-4.5	-4.4
5-Hydroxymethylfurfural	237332	-5.2	-4.3	-5.0
2 (5H)-Furanone	10341	-4.0	-4.0	-4.1
Cyclopentanone	8452	-4.3	-3.6	-4.0
Compounds	PUBCHEM I.D.	RET Affinity (kcal/mol)	PIK3CA Affinity (kcal/mol)	TCTN3 Affinity (kcal/mol)
