# Supplementary material for: Investigation of biometabolites and novel antimicrobial peptides derived from promising source Cordyceps militaris and effect of non-small cell lung cancer genes computationally
Source: PLoS One. 2025 Jan 23;20(1):e0310103. doi: 10.1371/journal.pone.0310103 (PMC11756765; doi:10.1371/journal.pone.0310103)
Supplement: Attachment — Submitted filename: PlosOne-Manuscript,rev.docx [file pone.0310103.s007.docx]

RESEARCH ARTICLE

Investigation of Biometabolites and Novel Antimicrobial Peptides Derived from Promising Source *Cordyceps militaris* and Effect of Non-Small Cell Lung Cancer Genes Computationally

Neelum Shehzadi^1^, Mai Abdel Haleem A. Abusalah^2^, Naveed Ahmed^3^, Muhammad Afzal^1, *^, Sarmir Khan^4^, Yalnaz Naseem^5^, Noshaba Mehmood^1^ and Kirnpal Kaur Banga Singh^3, *^

^1^ Faculty of Science and Technology, Department of Basic and Applied Chemistry, University of Central Punjab, Lahore, Pakistan

^2^ Department of Medical Laboratory Sciences, Faculty of Allied Medical Sciences, Al-Ahliyya Amman University, Amman, Jordan

^3^ Department of Medical Microbiology and Parasitology, School of Medical Sciences, Universiti Sains Malaysia, Kubang Kerian 16150, Kelantan, Malaysia

^4^ The First Affiliated Hospital of Zhengzhou University & Institute of Reproductive Health, Henan Academy of Innovations in Medical Science, NHC Key Laboratory of Birth Defects Prevention , China

^5^ Institute of drug discovery and development, Zhengzhou University, Zhengzhou 450001, China

***Corresponding author**
Email: [dr.afzal@ucp.edu.pk](mailto:dr.afzal@ucp.edu.pk) (M.A); [kiren@usm.my](mailto:kiren@usm.my) (K.K.B.S.)

**Abstract**

Mushrooms are considered one of the safe and effective medications because they have great economic importance due to countless biological properties. *Cordyceps militaris* contains bioactive compounds with antioxidant, antimicrobial and anti-cancerous properties. This study was projected to analyze the potentials of biometabolites and to extract antimicrobial peptides and protein from the *C. militaris.* An in-vitro analysis of biometabolites and antimicrobial peptides was performed to investigate their pharmacological potentials followed by quantification and characterization of extracted protein. Computational analysis on non-small cell lung cancer genes (NSCLC) was performed on quantified compounds to interpret the biometabolites from *C. militaris* that could be potential drug candidate molecules with high specificity and potency. A total of 34 compounds representing 100% of total detected constituents identified were identified using GCMS analysis and 20 compounds using LC-MS which showed strong biological activities. FT-IR spectroscopy manifest powerful instant peaks to have different bioactive components including carboxylic acid, phenols, amines and alkanes present in methanolic extract of *C. militaris*. In *C. militaris*, higher protein concentration was observed in 70% concentration of protein extract (500 µg/ml ± 0.025) as compared to 50% (450 µg/ml ± 0.012) and 30% (250 µg/ml ± 0.07). The best antioxidant activity of methanolic extracts was 80a ± 0.03, antidiabetic activity was 37 ± 0.057 and anti-inflammatory activity was 40 ± 0.021 at 12 mg/ml. Antibacterial activity for 70% concentration of Cordyceps protein and methanol extract was seen against *Escherichia coli* (4 mm), *Klebsiella pneumoniae* (2.7 mm), *Staphylococcus aureus* (5.4), *Streptococcus viridans* (4.3 mm). Indolizine, 2-(4-methylphenyl) has most binding affinity (micromolar) and optimized properties to be selected as the lead inhibitor. It interacts favorably with the active site of RET gene of NSCLC and is neuroprotective and hepatoprotective.

**Keywords:** Antimicrobial peptides; DPPH; scavenging activity; Antibiotic resistance; MMPBSA analysis; Lung Cancer; GROMACS.

**Introduction**

Mushrooms are widely distributed throughout the earth biosphere (1). Mushrooms are the best substitute sources secondary metabolites includes anthraquinones, terpenes, steroids, derivatives of benzoic acid, as well as contains few primary metabolites with proteins, peptides, and oxalic acid (2). Mushrooms possess various pharmacological importance including antibacterial, radical scavenging, immunomodulating, antitumor, anti-hypercholesterolemic, cardiovascular, antifungal, antidiabetic, hepatoprotective, anti-parasitic and detoxification effects (3). Mushrooms are abundantly used in medications and food. Mushroom extracts are valuable against cancer treatment (4). Because mushrooms comprise antitumor activities and other properties for treatment of various chronic diseases.

Most of the studies have been conducted on the other species of Cordyceps rather than *Cordyceps militaris* (5). There is no current literature reported on GC-MS and LC-MS interpretation of methanolic extract of *C. militaris* and isolation of antimicrobial peptides by treatment with proteolytic enzyme. The *C. militaris* is valuable medicinal and edible fungus with rich source of protein, cordycepin, polysaccharides and other bioactive compounds with antioxidant, antimicrobial and anti-cancerous properties (6). *Cordyceps militaris* is the complex composed of the fruiting body (the grass part), and the sclerotium (the dead body of insect), with the source of biologically active compounds that studied in nutraceuticals, biotechnology and therapeutic applications (7) and with the functions of improving, immunity, anti-inflammatory, antibacterial, blood pressure and lowering blood glucose. (8). Different bioactive compounds which includes polysaccharides, adenosine derivatives (mostly cordycepin), mannitol, ergosterol, protein and peptides are present in fruiting bodies and mycelia of *C. militaris* (9). *Cordyceps militaris* show variety of health traits which includes anti-inflammatory and antioxidant properties, reduce physical fatigue, help to manage blood glucose and to treat chronic kidney disease, improves liver functions and boost physical performance, regulation of blood pressure and beneficial uptake of oxygen in the body and also have antitumor and antiaging properties (10).

SDS-PAGE is an analytical method used for the protein separation on the basis of their molecular mass in an electric field (11). SDS act as detergent and give the negative charge to denature the proteins. Polyacrylamide gel is used to give the matrix or support to run the gel. Proteins move on the basis of their charge to mass ratio. Small size proteins move speedily rather than large size proteins (12).

An oxidant is a molecule that inhibits the oxidation process. When a chain reaction takes place in the cell, it causes death or damage to the cell (13). Antioxidants stop these chain reactions in a cell by removing free radicals and inhibiting different oxidation reactions (14). Dietary antioxidants play an essential role in preventing the oxidation reaction of lipids or cellular compounds by inhibiting chain reactions in cells (15).

The antidiabetic activity is also exhibited by the *C. militaris* (16). The main source of glucose in the blood is the hydrolysis of starch. In small intestines, only monosaccharides such as fructose and glucose can absorb from our diet, so polysaccharides in our diet need to be broken down into monosaccharides (17). The α-amylase enzyme hydrolyzes the oligosaccharides linkage of α-1,4-glycosidic and further breaks down disaccharides into monosaccharides (simple sugar) (18). α-glucosidase generates glucose from the non-reducing ends of oligosaccharides (19). The main inhibitor of α-glucosidase is acarbose which is commonly used to decrease glucose production by inhibiting this enzyme in small intestines (20). Researcher conducted and evaluate the antimicrobial activities of *C. militaris* at different concentrations against bacterial and fungi strains (21) (22).

The present work was planned to identify and characterize biological active compounds from fraction based on polarities of methanolic crude extract of *C. militaris* and to isolate the mushroom bioactive peptides (MBAPs) from the *C. militaris*. Different protocols and different strains were used to check out the activity of biometabolites and peptides against bacteria and with the DPPH, α-amylase and BSA assay to find out the antioxidant, anti-diabetic and anti-inflammatory effect respectively and the characterization by SDS-PAGE. In this study we investigated biometabolites and antimicrobial peptides from medicinal mushroom to eliminate the risks of microbial infections and other ailments. Computation analysis on non-small cell lung cancer genes (NSCLC) was performed by GC-MS quantified compound to evaluate the biometabolites from *C. militaris*.

**Materials and methods**

**Sample Collection**

The fresh fruiting bodies of medicinal mushroom *C. militaris* was purchased from the house of Mushroom, Lahore. The mushroom was taxonomically classified, identified and confirmed from the University of Central Punjab, Lahore Pakistan.

**Preparation of Methanol Extract of *Cordyceps militaris***

**Sample preparation**

Fresh fruiting bodies of *C. militaris* was rinsed with distilled water to detach the contamination. The dried fruiting bodies of *C. militaris* were taken in a pestle and mortal. By adding liquid nitrogen, the fruiting bodies of mushroom was crushed till it turns into powder form by lysis its cell wall. Finally, the dried sample was crushed into fine powder.

**Extraction Method**

The mushroom sample was soaked in methanol and this suspension was incubated in shaking incubator for the duration of 10 days. After the assigned period, the sample mixture was filtered through cheese cloth and then further by Whatman filter paper. At 50 ºC, on rotary evaporator, filtrate was further concentrated. The procedure was repeated until the desired semi-solid sample is obtained. The sample was collected and well-kept-up in air tight glass tubes. At the end, the methanol extract of fruiting bodies of *C. militaris* were ready for further analysis of the biological activities.

**Fourier Transform Infrared Spectroscopy (FT-IR)**

The methanol extract of *C. militaris* were mixed up in KBr salts and converted to fine pellet using a pestle and mortal. For spectroscopy analysis, two FT-IR spectrometers with 4000 and 500 cm^-1^ scan range were used.

**Gas Chromatography-Mass Spectrometry**

GC-MS (Model: 7890B, 5977A, Agilent USA) was used to evaluate the whole methanolic extract of the *C. militaris.* A DB-5 capillary tube with 30 m length, 0.25 µm film thickness and 0.25 mm diameter was used to investigate the biometabolites of methanolic extract using a Hewlett-Packard model 6890 working ionization energy at 75 eV. The carrier gas was helium, which was used a rate of 1 ml/minute. MS source was set at temperature to 280°C, the split ratio to 1:6, injected 1 µL of sample extract down the column. In order to interpret and analyze the components after separation in the column at 75 eV, FID spectroscopy was used. In NIST library, the spectra of the unknown components to known components were compared.

**Liquid Chromatography-Mass Spectrometry**

LC-ESI-MS which constitutes the triple quadrupole liquid chromatography-mass spectrometer (Finnigan TSQ Quantum Ultra EMR, Thermo Scientific) was used to analyze the methanolic extract of *C. militaris*. Components can be separated by Liquid Chromatography and charge ions identified by the Mass Spectrometer (MS). The chemicals were separated chromatographically using a Shim-pack XR-ODS III column. The mass spectrometer was set to Multiple Reaction Monitoring mode, and the fragmentation patterns of the standards were analyzed by injecting each standard solution at 0.1 g/mL directly into mass spectrometer. By comparing mass spectrometry fragmentation patterns and their retention time to those of legitimate standards, the major components of *C. militaris* were discovered

**Biological Activities**

**Antioxidant activity (DPPH)**

The radical scavenging of DPPH was done by the procedure given by (23) with modification. Different concentration of extracts and ascorbic acid (12, 6, 3, 1.5, 0.75 mg/mL) of 1 mL were mixed with 1 mL of 90 µM DPPH in dark. It was then incubated for 30 minutes at room temperature. Positive control was ascorbic acid while DPPH with methanol was used as negative control. Triplicates were run for the result at different time intervals.

% Radical Scavenging activity = $\frac{Blank absorbance-Sample absorbance}{Blank absorbance}$ $\times$100

**Antibacterial activity**

Antibacterial activity of methanol extract of *C. militaris* was investigated by disc diffusion method in which 4 bacterial strains, two Gram-positive bacteria (*Streptococcus viridans* and *Staphylococcus aureus*) and two Gram-negative bacteria (*Klebsiella pneumoniae* and *Escherichia coli*) were used. Nutrient broth was used for culture preparation. Hundred ml of nutrient formed by dissolving 1 g of peptone, 1 g NaCl and 0.5 g yeast mixed in 100 ml of distilled water in a flask and autoclave at 121°C for 1 hour. The mixture was poured into different test tubes and 100 µl of bacterial strains is added to each test tube and labeled accordingly. It was then incubated for 24 hours in a shaking incubator (24). For the preparation of agar plates, 10 g of nutrient agar was disintegrating in 250 mL of distilled water in a conical flask. Autoclaved at 121°C for 1 hour. The agar mixture was then added to autoclaved plates for a few minutes.

**Disc Diffusion Method**

Microorganism suspension is prepared and poured on a solid Muller Hinton agar media plate. Sterilized discs were dipped in methanol extract of 200 mg/ml concentration and placed on a media plate. The plates were wrapped tightly with parafilm and placed on an incubator for 24 hours at 37ºC. Positive control is Cefotaxime while negative control is DMSO. The zone of inhibition was calculated as mm (25).

**Antidiabetic activity (DNSA method)**

In-vitro antidiabetic activity was performed using DNSA method by protocol given by (26). Different concentrations (12, 6, 3, 1.5, 0.75 mg/ml) were devised. Take 5 µl of α–amylase was treated with 195 µl of methanolic extract of *C. militaris.* Incubate the sample at 37°C for 10 minutes. After incubation, add 50 µl of 1 % starch solution, incubate for 10 minutes. Then added 50 µl of DNSA solution and further incubate it for 10 minutes. Absorbance was checked by ELISA at 630 nm. Negative control is distilled water while metformin exploited as a standard. Test was performed in triplicate at different time intervals.

% Inhibition = $\frac{Control absorbance-Sample absorbance}{Control absorbance}$ $\times$100

**Anti-inflammatory activity**

The effect of sample extract against anti-inflammatory activity was checked by the method reported by (27) with little modifications. Different concentration of sample extracts of 12, 6, 3, 1.5, 0.75 mg/ml were devised and 100 µl of sample extract was taken in centrifuge tubes and add 0.5 ml of BSA. The mixture was incubated for 20 minutes at 37ºC and then retained on a water bath for 10 minutes at 70°C. Take absorbance reading at ELISA reader at 630 nm in Elisa plates. The % BSA inhibition was intended by the formula given below.

% BSA Inhibition = $\frac{Control absorbance-Sample absorbance}{Control absorbance}$ $\times$100

**Extraction of Peptides**

About 50 g of dried fruiting bodies of *C. militaris* were treated with liquid nitrogen and squashed in chilled pestle and mortal. Phosphate buffer saline (PBS) was added into the crushed fruiting bodies after a minute with pH 7.4. Freeze the sample at -20ºC for further liquification and procedure was repeated for another cycle (28). Sample hence obtained was centrifuged at 10000 rpm for 15 to 20 minutes and segregate the supernatant. Supernatant was stored at -20ºC for next procedure.

**Salting in and Salting Out**

The ammonium sulfate treatment was used to precipitate out the supernatant. Thirty, 50, and 70% ammonium sulfate precipitation were used for salting-in (29). To remove salt, we poured the sample onto a dialysis membrane, sealed it on both sides, and set it on a magnetic stirrer for 12 hours. Afterwards, the sample was spun at 6,000 rpm for ten to fifteen minutes. The resulting pellet was stored in PBS buffer at -20°C for future use (30).

**Protein Quantification and Characterization**

**Bradford assay**

To quantify the unknown protein concentration, at first a standard curve was generated using BSA as a standard and making its dilutions for obtaining different concentrations at different levels. Stock solution of BSA is prepared that was 1 mg/mL and then dilutions of 10, 20, 40, 60, 80, 100 µl were devised. The absorbance was measured using an ELISA reader. In each well of the 96-well Elisa plate, add 250 µl of Bradford reagent and 10 µl of either the BSA standard or the unknown sample fractions. Every sample and standard was run three times to determine the absorbance at 630 nm. More intense the blue color, more concentration of protein is present. In order to determine the total protein content of the unknown sample, the absorbance readings were compared to the standard curve (31).

**Characterization of protein**

The samples were prepared for the SDS-PAGE to characterized the protein. The Protein ladder used for SDS-PAGE was Thermo Fisher Scientific pre-stained protein ladder. The protein range was 10 – 180 kDa which was the mixture of ten pre-stained recombinant proteins with different chromophores (32).

**Biological activities for Cordyceps protein**

**Antimicrobial activity**

Antimicrobial assay of mushroom sample was performed. Two strains of bacteria were used gram positive bacteria *Staphylococcus aureus* and *Streptococcus viridans* and Gram-negative bacteria *Escherichia coli* and *Klebsiella pneumoniae.*

Nutrient broth was used for culture preparation. 100 mL of nutrient formed by dissolving 1 g of peptone, 1 g NaCl and 0.5 g yeast mixed in 100 mL of distilled water in a flask and autoclave d at 121°C for 1 hour. The mixture was poured into different test tubes and 100 µL of bacterial strains is added to each test tube and labeled accordingly. It was then incubated for 24 hours in a shaking incubator.

A conical flask containing 250 ml of distilled water and 10 g of disintegrated nutritional agar was autoclaved at 121°C for 1 hour in order to prepare agar plates. The agar mixture was then added to autoclaved plates for a few minutes.

**Disc diffusion method**

Placed the culture medium and poured petri plates and labelled the plates in biosafety cabinet. By using culture swab, spread the bacterial culture into petri plates. Antibiotic discs were dipped into 100 µl of protein extract and placed on a media plate. The plates were wrapped tightly with parafilm and placed in an incubator for 24 hours at 37°C for bacterial growth. Partial peptides having antimicrobial activity showed clear zone of inhibition. For positive control as Cefotaxime was used while PBS taken as negative control.

**Insilico Analysis**

Effect of GC-MS Quantified Compounds on RET gene at Non-Small Cell Lung Cancer

**Ligand Retrieval**

A methanolic crude extract of *C. millitaris* was recovered as a ligand by GC-MS. The chemicals were isolated and studied. 3D structure of all compounds was obtained from PubChem database.

**Target Protein Selection**

Targeted susceptible protein was identified from the literature. 3D structure of targeted protein was retrieved from the PDB database (https://[www.rcsb.org](http://www.rcsb.org)) (Accessed on 1 September 2023). 3D structure of NSCLC gene of RET (PDB ID: 6NEC), PIK3CA (PDB ID: 7L1B), and TCTN3 (PDB ID: 7QRX) was downloaded from PDB database.

**Molecular Docking**

AutoDoc Vina was used to performed docking for RET (PDB ID: 6NEC) and phytocompounds (ligands) of *C. militaris* through GC-MS analysis. PubChem (<http://pubchem.ncbi.nlm.nih.gov/>) accessed on 3 September 2023 download ligands in SDF format. 3D molecular structure of protein with unique PDB ID was downloaded in PDB format from Protein Data Bank (PDB). While, discovery studio software (Version 2021) was used to prepare the target protein (33).

**Lead Optimization**

Lead optimization was used to enhance the ligand’s binding affinity, pharmacokinetic properties, selectivity and safety profiles. It was performed through the BioSolveIT software suite. Energy minimization was performed in UCSF Chimera (version 1.17.1) using the Gasteiger approach (Gasteiger & Marsili, 1978) to get a zero net charge on the ligands. Following the optimization process, the ligands that were chosen underwent a conversion into a ‘mol2’ file format in order to facilitate molecular docking and subsequent evaluation.

**Pharmacokinetics properties**

SwissADME was used to characterize the highest binding energy compound against NSCLC genes. It was used to evaluate the pharmacokinetics, drug-likeness of the drug, lipophilicity, along with its solubility, and toxicity (34).

**Toxicity screening (ProTox-II)**

For predicting various toxicity endpoints, such as hepatotoxicity, carcinotoxicity, immunotoxicity, cytotoxicity, toxicity targets and their adverse outcome pathways, Protox-II was used.

**Molecular Dynamic Simulation**

For conducting high-performance molecular dynamics simulations, GROMACS version 2023 which is accessed at the web address: <https://www.gromacs.org/>. It operates by simulating the depiction of intricate domain motions within macroscopic molecules. It was used to investigate various ligand structural conformations residing inside the binding pocket while keeping fixed particular position of other molecules or amino acids or conformational fluctuations that may occur during molecular simulations and gives the stability insights of interaction between protein and ligand.

**MMPBSA Analysis**

The Molecular Mechanics Poisson Boltzmann Surface Area (MMPBSA) was manipulated in screening step for post-refinement and predicted free binding energy calculation G_binding_ by using GROMACS version 2023. The outcomes derived from the MMPBSA computations encompassed Binding Free Energy (BFE) values. The results are widely acknowledging as very precise outcomes in the realm of computational analysis for protein-ligand complexes simulated insilico. In this analysis, electrostatic forces, van der walls interactions, molecular mechanics potential energy and free energy of solvation, including polar and non-polar interactions were involved in the analysis of binding free energy (BFE).

MMPBSA methods used the following equations in order to interpretation of protein and ligand binding free energy by using GROMACS version 2023.

(i)	$\Delta$G*_bind_* _=_ G_c_*_omplex_* $-$ (G*_protein_* _+_ G*_ligand_*)

(ii)	G*_x_* = < E*_MM_* > $-$TS + < G*_solvation_* >

(iii)	E*_MM =_* E*_bonded_* _+_ *E_non-bonded_* _=_ E*_bonded_* _+_ (E*_vdw_* + E*_elec_*)

(iv)	G*_solvation_* _=_ G*_polar_* + G*_non-polar_*

(v)	G*_non-polar_* = $\gamma$SASA + *b*

Where in Eq. (i), G*_complex_* is the whole complex free energy while the G*_protein_* and G*_ligand_* are the protein and ligand’s total free energy respectively. In Eq. (ii) and (iii) G*_x_* is the free energy for the individual species, E*_MM_* is the vacuum molecular mechanics’ potential energy and it is the sum of bonded, electrostatic interaction and van der Waals potentials. TS shows the binding free energy entropic contribution, where T is the temperature and S indicates the entropy, and G*_solvation_* depicts the solvation free energy for both polar and non-polar solvation free energy. In Eq. (iv) G*_polar_* demonstrated the electrostatic free energy solvation while G*_non-polar_* is the non-electrostatic free energy solvation. In Eq. (v) SASA indicates the solvent-accessible surface area while $\gamma$ and *b* are the empirical constant.

**Statistical Analysis**

Each result is expressed as a mean and standard error of measurement. The difference between treated group and control group was compared using one-way ANOVA followed by Dunnett’s post hoc test. The analysis was carried out by using SPPSS and significant p > 0.05 were considered.

**Results**

**Fourier Transform Infrared Spectroscopy**

FTIR spectroscopy manifest to have different bioactive components, including carboxylic acid, phenols, amines and alkanes present in methanolic extract of *C. militaris*. Eight functional groups identified from the methanol extract are shown in **Table 1** and **Figure 1**. The powerful instance peak with the O-H stretching at 3323 cm^-1^ were observed.

**Table 1.** **FTIR interception of components of methanol extract of *C. militaris.***

| **Sr. no.** | **Wavenumber cm^-1^** | **Functional group** | **Compound Class** |
| --- | --- | --- | --- |
| 1 | 3323.22 | O-H stretching | Alcohols, phenols |
| 2 | 2944.82 | C-H stretching | Alkane |
| 3 | 2833.03 | C-H stretching | Alkane |
| 4 | 1655.20 | N-H bending | Amine |
| 5 | 1449.05 | O-H bending | Carboxylic acid |
| 6 | 1112.57 | C-N stretching | Amine |
| 7 | 1020.50 | C-N stretching | Amine |
| 8 | 611.07 | C-H bending | Alkane |

**Gas Chromatography- Mass Spectrometry**

GC-MS analysis shows the chemical composition of methanolic extract of *C. militaris* in **S1** **Figure**. Thirty-four compounds show 100% of total detected constituents were identified and the GC-MS analysis. The major compounds were 1,2,3-Benzenetriol (21.77), 5-Hydroxymethylfurfural (19.61), Methyl 3,4,5-trihydroxybenzoate (5.45), 2(5H)-Furanone (3.70), 2-Furancarboxylic acid (2.65) shown in **S1** **Table**.

**Liquid Chromatography-Mass Spectrometry**

Twenty compounds representing 100% of total detected constituents were identified and the major compounds were malonic acid, 1,2,3-Trihydroxybenzene, 5-Ethoxy-4,5-dihydro-2(3H)-furanone, Caftaric acid, 4-Ethoxy-4-oxobutanoic, Oxoadipic acid, Trans-Aconitic acid, 3-Methyl-4-hydroxy-5-all-trans-heptaprenylbenzoate and Ampeloside Bs1 as shown in **S2 Table**.

**Biological activities of methanolic extract of *Cordyceps mlitaris***

**Antioxidant activity**

The antioxidant activity of *Cordyceps militaris* of methanolic extract fractions at different concentrations (12, 6, 3, 1.5, 0.75 mg/ml) were assessed by DPPH radical scavenging assay. The result indicated that methanolic extract of *Cordyceps militaris* show a high percentage of inhibition (80 ± 0.03) at 12 mg/ml concentration and lowest (55 ± 0.09) at 0.75 mg/ml concentration as manifested in **Table 2**. The free radical scavenging ability of *C. militaris* varies remarkably (p ˂ 0.005).

**Table 2. DPPH Percentage radical scavenging activity of methanol extract of *C. militaris.***

| **Fractions** | **Concentrations**  **(mg/mL)** | **DPPH % Inhibition**  **(Mean ± S.D.)** | **IC_50_** |
| --- | --- | --- | --- |
| Methanol | 0.75 | 55 ± 0.09 | 2.71 |
|  | 1.5 | 60 ± 0.05 |  |
|  | 3 | 61 ± 0.02 |  |
|  | 6 | 76 ± 0.04 |  |
|  | 12 | 80 ± 0.03 |  |
| Ascorbic acid | 0.75 | 80 ± 0.07 | 3.89 |
|  | 1.5 | 83 ± 0.06 |  |
|  | 3 | 85 ± 0.03 |  |
|  | 6 | 89 ± 0.02 |  |

The values were the average of triplicate samples (n =3) ± S.D., the results are significant because two-way ANOVA shows (p ≤ 0.05).

**Antimicrobial activity**

The results obtained for antibacterial activity are shown in **Table 3**. In *Escherichia coli*, methanol extract manifests the maximum zone of inhibition (6.9 ± 0.04). While (6.5 ± 0.01), (3 ± 0.98) and (3.2 ± 0.42) zone of inhibition against bacterial strains *Klebsiella pneumonia, Streptococcus viridans,* and *Staphylococcus aureus* respectively **(S2 Figure)**. Positive control is Cefotaxime. While negative control is DMSO. The ability of inhibition of bacterial strains of methanol extract of *C. militaris* varies significantly (p ˂ 0.05).

**Table 3. Antibacterial activity of methanol extract of *C. militaris* against bacterial strains.**

| **Bacterial strains** | **Fractions**  **(mg/ml)** | **Zone of inhibition (mm)**  **Mean ± S.D** |
| --- | --- | --- |
| *Escherichia coli* | Methanol | 6.9 ± 0.04 |
|  | Cefotaxime | 11 ± 0.85 |
| *Klebsiella pneumoniae* | Methanol | 6.5 ± 0.01 |
|  | Cefotaxime | 10.2 ± 0.34 |
| *Streptococcus viridans* | Methanol | 3 ± 0.98 |
|  | Cefotaxime | 6 ± 0.34 |
| *Staphylococcus aureus* | Methanol | 3.2 ± 0.42 |
|  | Cefotaxime | 11.4 ± 0.65 |

The values were the average of triplicate samples (n =3) ± S.D., the results are significant because two-way ANOVA shows (p ≤ 0.05).

**Antidiabetic activity**

The antidiabetic activity of methanolic extract of *Cordyceps militaris* at different concentrations (12, 6, 3, 1.5, 0.75 mg/ml) was analyzed as shown in **Table 4**. The highest antidiabetic activity was shown (37 ± 0.057) at 12 mg/ml and lowest activity (13 ± 0.084) at 0.75 mg/ml. The percentage inhibition of antidiabetic activity of *C. militaris* is significantly p ˂ 0.05.

**Table 4. Antidiabetic activity of methanol extract of *C. militaris.***

| **Fractions** | **Concentrations**  **(mg/mL)** | **% Inhibition of α-Amylase**  **(Mean ± S.D)** | **IC_50_** |
| --- | --- | --- | --- |
| Methanol | 0.75 | 13 ± 0.084 | 4.26 |
|  | 1.5 | 19 ± 0.089 |  |
|  | 3 | 21 ± 0.046 |  |
|  | 6 | 34 ± 0.023 |  |
|  | 12 | 37 ± 0.057 |  |
| Ascorbic acid | 0.75 | 34 ± 0.029 | 1.17 |
|  | 1.5 | 38 ± 0.087 |  |
|  | 3 | 42 ± 0.056 |  |
|  | 6 | 45 ± 0.019 |  |

The values were the average of triplicate samples (n =3) ± S.D., the results are significant because two-way ANOVA shows (p ≤ 0.05).

**Anti-inflammatory activity**

To assess the anti-inflammatory mechanism of *C. militaris*, its methanolic extract capability to inhibit bovine serum denaturation is calculated at different concentrations (12, 6, 3, 1.5, 0.75 mg/ml) shown in **Table 5**. It was observed that methanolic extract shows maximum inhibition of denaturation of BSA (40 ± 0.021) which is less than standard diclofenac (86 ± 0.096) at 12 µl/ml concentration. The result shows a concentration-dependent on inhibition of protein denaturation by *C. militaris.* The ability to protein inhibition (BSA) of *C. militaris* varies significantly (p ˂ 0.05).

**Table 5. Anti-inflammatory activity of methanol extract of *C. militaris.***

| **Fractions** | **Concentrations**  **(mg/mL)** | **BSA inhibition**  **(Mean ± S.D.)** | **IC_50_** |
| --- | --- | --- | --- |
| Methanol | 0.75 | 23 ± 0.097 | 1.75 |
|  | 1.5 | 27 ± 0.070 |  |
|  | 3 | 34 ± 0.035 |  |
|  | 6 | 38 ± 0.089 |  |
|  | 12 | 40 ± 0.021 |  |
| Ascorbic acid | 0.75 | 45 ± 0.096 | 1.48 |
|  | 1.5 | 48 ± 0.091 |  |
|  | 3 | 53 ± 0.098 |  |
|  | 6 | 80 ± 0.012 |  |

The values were the average of triplicate samples (n =3) ± S.D., the results are significant because two-way ANOVA shows (p ≤ 0.05).

**Protein Quantification**

**Bradford Method**

Total protein quantification was done by Bradford assay. Unknown protein concentration sample was found out by comparing the sample absorbance with the standard curve. Different concentrations of BSA were used and its standard curve was obtained as shown in **Figure 2A**.

Protein concentration calculated from Bradford assay was summarized as the 70% concentration showed higher protein quantity which was 500 µg/ml. While 30% and 50% showed the 250 µg/ml and 400 µg/ml respectively as manifested in **Table 6**.

**Table 6. Total protein concentration in different fractions of *C. militaris.***

| Sr. no. | Fraction (%) | Concentration (µg/ml) |
| --- | --- | --- |
| 1 | 30 | 250 µg/ml ± 0.08 |
| 2 | 50 | 400 µg/ml ± 0.010 |
| 3 | 70 | 500 µg/ml ± 0.030 |

**Characterization of protein**

**SDS-PAGE**

The sample were subjected to SDS-PAGE to find out the molecular weight of the protein as shown in **Figure 3A**. Thermo Fisher Scientific pre-stained Protein Ladder was used which ranging from ~10 to ~180 kDa manifested in **figure 3B**. The sample with 70% shows the maximum bands compared to the 50 and 30% ranges from ~100 to ~10 kDa. 70% showed cluster of bands with size of 70 kDa, 64 kDa, 59 kDa, 45 kDa, 32 kDa, 22 kDa. 50% showed weak bands with the size of 40 kDa, and 22 kDa. while 30% showed no band.

**Proteolytic enzyme treatment**

Protein isolated from *Cordyceps militaris* treated with proteolytic enzyme (i.e., pepsin, trypsin and papain) in order to make the peptides depicted in **Figure 3C**. The purpose of using proteolytic enzymes to check whether Cordyceps protein of higher kDa digested to convert into lower kDa antimicrobial peptides. The optimum temperature and pH for pepsin is 37ºC and 2-4 respectively (35) and for trypsin the optimum temperature and pH is 65ºC and 9 respectively (36). For papain, the optimum temperature and pH is 37-80ºC and 7 respectively (37). Thermo Fisher Scientific pre-stained Protein Ladder was used ranging from ~10 to ~180 kDa. SDS-PAGE analysis of the proteolytic enzymes treated extract showed only trypsin digestion and show maximum peptides in lower kDa between 15-10 kDa.

**Biological activities of *Cordyceps militaris* protein**

**Anti-bacterial activity**

Anti-bacterial activity was performed for identifying the peptides activity from the fruiting bodies of *C. militaris* at different concentrations and zone of inhibition measured in mm, as shown in **Table 7**. Fractions of 70%, 50% and 30% showed visible zone of inhibition i.e., (4 ± 0.22), (3.5 ± 0.06) and (3.6 ± 0.01) in *Escherichia coli* respectively. In *Klebsiella pneumonia,* 70%, 50%, and 30% fraction showed the (2.7 ± 0.06), (0.5 ± 0.09) and (0.2 ± 0.03) zone of inhibition respectively.

**Table 7. Antibacterial activity of *C. militaris*.**

| **Bacterial strains** | **Fractions** | **Zone of inhibition (mm)**  **Mean ± S.D** |
| --- | --- | --- |
| *Escherichia coli* | 30% | 3.6 ± 0.01 |
|  | 50% | 3.5 ± 0.06 |
|  | 70% | 4 ± 0.22 |
|  | Cefotaxime | 11 ± 0.09 |
| *Klebsiella pneumoniae* | 30% | 0.2 ± 0.03 |
|  | 50% | 0.5 ± 0.09 |
|  | 70% | 2.7 ± 0.06 |
|  | Cefotaxime | 9.4 ± 0.07 |
| *Streptococcus viridans* | 30% | 4 ± 0.176 |
|  | 50% | 3.3 ± 0.07 |
|  | 70% | 4.3 ± 0.034 |
|  | Cefotaxime | 6 ± 0.10 |
| *Staphylococcus aureus* | 30% | 1 ± 0.04 |
|  | 50% | 3 ± 0.02 |
|  | 70% | 5.4 ± 0.08 |
|  | Cefotaxime | 6 ± 0.07 |

The highest antibacterial activity found against *Staphylococcus aureus* which showed the maximum zone of inhibition in 70% i.e., (5.4 ± 0.08), while 50% and 30% was (3 ± 0.02) and (1 ± 0.04) respectively. In *Streptococcus viridans,* 70%, 50% and 30% fraction showed the (4.3 ± 0.034), (3.3 ± 0.07) and (4 ± 0.176) zone of inhibition respectively. Positive control is Cefotaxime. Negative control is PBS. The ability of inhibition of bacterial strains of protein fractions of *C. militaris* varies significantly (p ˂ 0.05).

**Effect of GC-MS Quantified Phytoconstituents on Non-Small Cell Lung Cancer (NSCLC) Genes**

**Protein Retrieval**

3D structure of targeted protein of NSCLC i.e., RET gene (PDB ID: 6NEC), PIK3CA (PDB ID: 7L1B), and TCTN3 gene (PDB ID: 7QRX) was downloaded from Protein DataBank (PDB).

**Active Sites Prediction**

DeepSite tool were performed to predict the binding pockets of RET gene (PDB ID: 6NEC) which involve in causing NSCLC disease. Five main binding sites were identified and pockets highlighted by yellow arrows as shown in **Figure 4A**. The binding positions and their score are shown in **S3** **Table** respectively.

**Molecular Docking**

AutoDoc Vina was used which determined the binding energies and the highest binding scores obtained by Indolizine, 2-(4-methylphenyl)- in NSCLC at RET gene (PDB ID: 6NEC) while second highest binding energy in PIK3CA (PDB ID: 7L1B), and TCTN3 gene (PDB ID: 7QRX) as compared to RET gene as shown in **S4 Table**. Figure 4B, 4C reveal that the compounds' binding interactions were mostly caused by alkyl forces in the evaluated compounds and pi-interactions between the ligand and non-small cell lung cancer protein.

**Molecular Dynamic Simulation**

**Molecular Dynamic Simulation (100ns)**

The molecular dynamics simulations for 100ns were performed with GROMACS version 2023, an open-source suite specifically intended for high-performance molecular dynamics simulations. The software can be accessed at the following web address: https://www.gromacs.org/.This particular service functions by replicating the representation of complex domain movements within macroscopic molecules. Moreover, it facilitates the exploration of potential structural alterations or conformational variations that may occur during molecular simulations, while providing valuable insights into protein-ligand interactions stability.

Molecular docking results indicate that among the several compounds examined, 2-(4-methylphenyl) Indolizine exhibited a superior dock score, accompanied by the highest cluster. The co-crystallized structure obtained from the X-ray structure is utilized for conducting a molecular dynamics (MD) simulation investigation. The dock score (-CDOCKER interaction energy, kcal/mol) of Indolizine, 2-(4-methylphenyl) was -8.0 kcal/mol with RET gene. The pose with the highest score within this cluster was selected as the optimal pose and subsequently subjected to Molecular Dynamics Simulations (MDS) in order to evaluate its stability for the whole duration of the simulation run as depicted in **Figure 4D**. The purpose of conducting Molecular Dynamics Simulations (MDS) was to get a deeper understanding of the behavior and movement of the ligand within the protein's binding pocket, as well as to investigate the manner in which the ligand adapts to its surroundings within the binding pocket.

**RMSD stability study of Indolizine, 2-(4-methylphenyl)**

The stability of the protein backbone (38), protein-ligand complex (com), and the ligand were thoroughly investigated during the simulation. The root-mean-square deviation (RMSD) values range from 0.4 nm to 0.6 nm for protein-ligand complex as depicted in **Figure 2B**. RMSD exhibited a deflection at around 65 nanoseconds for the systems under investigation. This deflection led to a little increase and subsequent drop in the RMSD of Indolizine, 2-(4-methylphenyl). The complex formed between the Indolizine molecule and 2-(4-methylphenyl)-RET was shown to be in a state of equilibrium and exhibited a consistent behavior, characterized by an average value of 0.63 nm. Significantly, RMSD of the complex exhibited increased oscillations from 65ns to 90ns, followed by a subsequent decrease and subsequent stability. The observed discrepancy is hypothesized to arise from the ligand's interaction with the protein's binding pocket. RMSD data indicate that the profiles were adequately equilibrated and have exhibited consistent stability.

**Protein backbone exhibited a compact conformation for 100ns simulation**

The radius of gyration (Rg) quantifies the degree of compactness shown by the protein, specifically with respect to its backbone structure. Rg for both systems was observed to fall within the range of 2.0 nm to 2.09 nm, respectively. This measurement provides insight into the protein's level of compactness during the simulation as depicted in **Figure 5A**. The calculated average radius of gyration (Rg) for Indolizine, 2-(4-methylphenyl) was determined to be 0.344 nm as illustrated in **Figure 5B**. The measured Rg profile of the reference exhibited an increase from 15ns to 35ns, which was also noted in the RMSD. Subsequently, the system underwent a thorough equilibration process during the simulation. In contrast, it was observed that the protein backbone of Indolizine, namely 2-(4-methylphenyl), exhibited a notably condensed conformation throughout the simulation, as seen by an average distance of 2.07 nm.

**Detection of RMSF with protein backbone**

The variations of the protein backbone residue atoms were assessed using the Root Mean Square Fluctuation (RMSF) method shown in **Figure 2C**. The residues of the backbone exhibit minimal variations. RMSF values of the residues were estimated to be less than 0.3 nm, indicating a high level of stability in the protein backbone. This finding is consistent with the results obtained from the root mean square deviation (RMSD) and radius of gyration (Rg) analyses. A slight elevation was seen in the residue LEU139 in comparison to the other residues, measuring around 0.49 nm with respect to the backbone and 0.57 nm with respect to the protein. Nevertheless, this residue is not present in close proximity to the binding pocket. The stability of Indolizine, 2-(4-methylphenyl), was assessed by examining the residues, namely those present in the active site. The distances between these residues were measured and found to be as follows: 0.102 nm for Asp193, 0.0816 nm for Ile189, 0.117 nm for Lys59, 0.0829 nm for Ser192, 0.0905 nm for Val105, 0.0752 nm for Leu182, 0.0672 nm for Gly111, and 0.255 nm for Leu131.

**Detection of Better Interaction Energy for protein-ligand complex**

The term "interaction energy" (IE) refers to the capacity of the ligand to bind to the active site protein. In this study, the IE was determined using two methods: Lennard-Jones interaction energy (LJIE) and Coulombic interaction energy (CIE). The CIE values for Indolizine, 2-(4-methylphenyl), have been seen to vary within the range of -84717.6 kJ/mol to -84887.4 kJ/mol, with an average value of -84322.4 kJ/mol. Additionally, the CIE plots for this compound exhibit a similar pattern to the plots of the calculated lowest LJIE for Indolizine, 2-(4-methylphenyl), showing a decrease in energy after 57000ps depicted in **Figure 5C**. The lower limit of the LJIE for Indolizine, namely 2-(4-methylphenyl), has been seen to range from -8236.42 KJ/mol to -8662.04 KJ/mol, with an average value of -8493.2 KJ/mol illustrated in **Figure 5D**. Based on the observed interaction energies, it is justifiable to infer that the ligand exhibits interaction with the protein.

**MMPBSA Analysis**

The MMPBSA analysis showed the calculation of free binding energy (G_binding_). The results depicted that the total binding free energy calculation involving the residues Asp193, Ile189, Lys59, Ser192, Val105, Leu182, Gly111, and Leu131 yields a value of -12.20 kcal/mol. This suggests a significant interaction between the protein RET and Indolizine, 2-(4-methylphenyl). Notably, the van der Waals interaction energy is found to be the highest, with a value of -25.35 kcal/mol, followed by the electrostatic energy with a value of -9.11 kcal/mol. These findings highlight the strong interaction between the key protein residues and the ligand.

**Preclinical Investigation**

SwissADME analysis showed that Indolizine, 2-(4-methylphenyl)- is the best anti-cancer compound against NSCLC as it has good lipophilicity Log p less than 4 Log P_o/w_ (2.90). The pharmacokinetic interpretation revealed good GI absorption, water solubility and abide by all the rules of Lipinski’s and does not violate it. According to pharmacokinetic analysis and compound interaction showed low glycoprotein permeability and CYP2C9 and CYP2C19 had no inhibitory effect. A BIOLED-EGG image of Indolizine, 2-(4-methylphenyl)- depicted in **Figure 6A** and Swiss Target Prediction are shown in **Figure 6B**.

**Toxicity screening**

The results depicted that there is low predictability against hepatoxicity, carcinogenicity, and immunotoxicity. The mutagenicity and cytotoxicity score are also very low and inactive. **Table 8** below illustrate that there is a high indication that Indolizine, 2-(4-methylphenyl)- will not be carcinogenic, mutagenic, or hepatotoxic.

**Table 8. Toxicity analysis of compound Indolizine, 2-(4-methylphenyl)- from Protox-II. Prediction status: Inactive.**

| **Toxicity Model Report of Drug** | | | |
| --- | --- | --- | --- |
| **Classification** | **Target** | **Shorthand** | **Probability** |
| Organ toxicity | Hepatotoxicity | Dili | 0.84 |
| Toxicity end points | Carcinogenicity | Carcino | 0.74 |
|  | Immunotoxicity | Immune | 0.78 |
|  | Mutagenicity | Mutagen | 0.67 |
|  | Cytotoxicity | cyto | 0.60 |
| Tox21-Nuclear receptor signalling pathways | Androgen Receptor (AR) | nr_ar | 0.97 |
|  | Aryl hydrocarbon Receptor | nr_ahr | 0.90 |
|  | Aromase | nr_aromase | 0.96 |
|  | Androgen Receptor Ligand Binding Domain (AR- LBD) | nr_ar_lbd | 0.99 |
|  | Estrogen Receptor Ligand Binding Domain (ER-LBD) | nr_er_lbd | 0.97 |
|  | Estrogen Receptor Alpha (ER) | nr_er | 0.92 |
|  | Nuclear factor (erythroid-derived 2)-like 2/antioxidant responsive element (nrf2/ARE) | sr_are | 0.98 |
|  | Peroxisome Proliferator Activated Receptor Gamma (PPAR-Gamma) | nr_ppar_gamma | 0.98 |
|  | Mitochondrial Membrane Potential (MMP) | sr_mmp | 0.93 |
|  | Heat shock factor response element | sr_hse | 0.98 |
|  | ATPase family AAA domain-containing protein 5 (ATAD5) | sr_atad5 | 0.98 |
|  | Phosphoprotein (Tumor Suppressor) p53 | sr_p53 | 0.95 |

The green color represents inactive toxicity based on a likelihood value from 0 to 10. A higher number indicates less toxicity.

**Discussion**

Mushrooms are the reservoirs of important components which are used for drug development and synthesis. Mushrooms contain the compounds which are biologically active. The presence of various bioactive components can be used to treat the various cancer disease, diabetes and antimicrobial infections (39, 40). The antimicrobial peptides play a significant role in different field such as medicine, food, agriculture and aquaculture. Eatable mushrooms are considered one of the safe and effective medication. Most of the studies have been conducted on the other species of Cordyceps rather than *C. militaris* and there is no current literature reported on GC/MS and LC/MS analysis of methanolic extract of *C. militaris* and isolation of antimicrobial peptides by treating with digestive enzyme.

*Cordyceps militaris* have many potent compounds that are already reported (41) but there is no work available on biometabolites analysis for methanol extract. So, in this study we investigated the biometabolites of methanol extract and antimicrobial peptides from medicinal mushroom to eliminate the risks of microbial infections and other ailments. These biometabolites and antimicrobial peptides have potential of biological activities. Analysis of FTIR spectroscopy of current studies of methanolic extract of *C. militaris* was evaluated and different bioactive components were confirmed by FTIR spectroscopy, including phenols, carboxylic acid, amines and alkanes which supports the biologically active compounds identified through GC-MS and LC-MS. The powerful instance peak with the O-H stretching at 3323 cm^-1^ were observed. Previous study showed two strong absorption peaks at 3397 cm^-1^ and 3192 cm^-1^ with the O-H stretching vibrations (42). These peaks are characteristics absorption peaks for polysaccharides.

GC-MS analysis shows the chemical composition of methanolic extract of *C. militaris*. Thirty-four compounds show 100% of total detected constituents were identified. The major compounds were 1,2,3-Benzenetriol (21.77%), 5-Hydroxymethylfurfural (19.61%), Methyl 3,4,5-trihydroxybenzoate (5.45%), 2(5H)-Furanone (3.70%), 2-Furancarboxylic acid (2.65%). The chemical composition determined by us is different from other studies. Few studies conducted in Italy, Poland, Spain and Iran, respectively, reported the major components of *C. militaris* were 4,5-Diamino-6-hydroxypyrimidine (29.01), Cyclobutanol (14.5), Trans-Cinnamic acid (30.78), and 1,6-Anhydro-beta-D-glucopyranose (40.1) (43). In our study LC-MS analysis showed various bioactive compounds in methanol extract of *C. militaris* as malonic acid, 1,2,3-Trihydroxybenzene, 5-Ethoxy-4,5-dihydro-2(3H)-furanone, Caftaric acid, 4-Ethoxy-4-oxobutanoic acid, Oxoadipic acid, Trans-Aconitic acid, 3-Methyl-4-hydroxy-5-all-trans-heptaprenylbenzoate and Ampeloside Bs1 by comparison to retention time. According to LC-MS analysis reported in previous studies showed the seven phenolic acids (chlorogenic acid, protocatechuic acid, gallic acid, coumaric acid, and hydroxybenzoic acid) and four flavonoids (quercetin, naringenin, apigenin, and rutin) reported in methanol extract of *C. militaris* (44).

Research findings show the antioxidant potential of *C. militaris*. The antioxidant capacity of *C. militaris* was measured by using DPPH assay which in non-enzymatic assay. The scavenging activity was increased with the increased of time. The antioxidant activity of *C. militaris* of methanolic extract fractions at different concentrations (12, 6, 3, 1.5, 0.75 mg/mL) were assessed by DPPH radical scavenging assay, evaluating the hydrogen transfer or free radical scavenging ability of the methanolic extracts using DPPH reagent and we compared their activity to the standard ascorbic acid The result indicated that methanolic extract of *C. militaris* show a high percentage of inhibition (80 ± 0.03) at 12 mg/ml concentration and lowest (55 ± 0.09) at 0.75 mg/ml concentration. The free radical scavenging ability of *C. militaris* varies remarkably (p ˂ 0.005). These results are consistent with the results of a previous study (45).

In *Escherichia coli*, methanol extract manifests the maximum zone of inhibition (6.9 ± 0.04). While (6.5 ± 0.01), (3 ± 0.98) and (3.2 ± 0.42) zone of inhibition against bacterial strains *Klebsiella pneumoniae, Streptococcus viridans,* and *Staphylococcus aureus* respectively. While the protein Fractions of 70%, 50% and 30% showed visible zone of inhibition i.e., (4 ± 0.22), (3.5 ± 0.06) and (3.6 ± 0.01) in *Escherichia coli* respectively. In *Klebsiella pneumonia,* 70%, 50%, and 30% fraction showed the (2.7 ± 0.06), (0.5 ± 0.09) and (0.2 ± 0.03) zone of inhibition respectively. The highest antibacterial activity found against *Staphylococcus aureus* which showed the maximum zone of inhibition in 70% i.e., (5.4 ± 0.08), while 50% and 30% was (3 ± 0.02) and (1 ± 0.04) respectively. In *Streptococcus viridans,* 70%, 50% and 30% fraction showed the (4.3 ± 0.034), (3.3 ± 0.07) and (4 ± 0.176) zone of inhibition respectively. The ability of inhibition of bacterial strains of fractions of *C. militaris* varies significantly (p ˂ 0.05).

Current study showed the antidiabetic activity of methanolic extract of *C. militaris* at different concentrations (12, 6, 3, 1.5, 0.75 mg/mL). The highest antidiabetic activity was shown (37 ± 0.057) at 12 mg/mL and lowest activity (13 ± 0.084) at 0.75 mg/ml. The percentage inhibition of antidiabetic activity of *C. militaris* is significantly p ˂ 0.05. The protein denaturation is a source to find inflammation. Therefore, to assess the anti-inflammatory mechanism of *C. militaris*, its extract capability to inhibits bovine serum denaturation is calculated at different concentration (12, 6, 3, 1.5, 0.75 mg/mL). It was observed that methanolic extract shows maximum inhibition of denaturation of BSA (40 ± 0.021) which is less than standard diclofenac (86 ± 0.096) at 12 µl/ml concentration. The result shows a concentration-dependent on inhibition of protein denaturation by *C. militaris.* The ability to protein inhibition (BSA) of *C. militaris* varies significantly (p ˂ 0.05).

Protein extractions were quantified by Bradford assay. Result revealed that extract of 70% concentration contains higher protein concentration (500 µg/ml ± 0.025) than the concentrations of 30 and 50%. The 30% concentration contains lower amount of protein (250 µg/ml ± 0.07). while 50% concentration shows the (450 µg/ml ± 0.012). Protein quantity analysis is important for quality control. The method discovered by the Marion Bradford was based on the absorption of max light by the protein sample bound to the brilliant blue G-250 (46). The sample with 70% shows the maximum bands compared to the 50 and 30% ranges from ~100 to ~10 kDa. 70% showed cluster of bands with size of 70 kDa, 64 kDa, 59 kDa, 45 kDa, 32 kDa, 22 kDa. 50% showed weak bands with the size of 40 kDa, and 22 kDa. while 30% showed no band. Therefore, it may be inferred from both the prior and present research that *C. militaris* fruiting bodies include dynamic proteins. The different concentration of *C. militaris* extracts treated with digestive enzymes pepsin, trypsin and papain in order to produce the peptides. Enzymatic treatment was used for *C. militaris* isolates to form peptides. Pepsin (37 °C and pH 2-4), Trypsin (65°C and pH 9) and Papain (37-80°C and pH 7) enzyme was used to make the peptides of most abundant protein in the *C. militaris.* SDS-PAGE analysis of the enzyme treated extract showed only trypsin digestion and show peptides in lower kDa.

The results of the molecular docking studies indicate that among the several compounds examined, 2-(4-methylphenyl) Indolizine exhibited a superior dock score, accompanied by the highest cluster. The co-crystallized structure obtained from the X-ray structure is utilized for conducting a molecular dynamics (MD) simulation investigation. The dock score (-CDOCKER interaction energy, kcal/mol) of Indolizine, 2-(4-methylphenyl) was -8.0 kcal/mol with RET gene. The pose with the highest score within this cluster was selected as the optimal pose and subsequently subjected to Molecular Dynamics Simulations (MDS) in order to evaluate its stability for the whole duration of the simulation run. MD simulation was performed by using GROMACS.

The RMSD stability study of Indolizine, 2-(4-methylphenyl) showed the values of the protein-ligand complex range from 0.4 nm to 0.6 nm. The complex formed between the Indolizine molecule and 2-(4-methylphenyl)-RET was shown to be in a state of equilibrium and exhibited a consistent behavior, characterized by an average value of 0.63 nm. Significantly, RMSD of the complex exhibited increased oscillations from 65ns to 90ns, followed by a subsequent decrease and subsequent stability. Radius of gyration (R_g_) quantifies the degree of compactness shown by the protein, specifically with respect to its backbone structure. R_g_ for both systems was observed to fall within the range of 2.0 to 2.09 nm, respectively. The calculated average radius of gyration (Rg) for Indolizine, 2-(4-methylphenyl) was determined to be 0.344 nm. The measured Rg profile of the reference exhibited an increase from 15ns to 35ns, which was also noted in the RMSD. Subsequently, the system underwent a thorough equilibration process during the simulation. In contrast, it was observed that the protein backbone of Indolizine, namely 2-(4-methylphenyl), exhibited a notably condensed conformation throughout the simulation, as seen by an average distance of 2.07 nm.

The variations of the protein backbone residue atoms were assessed using the RMSF method. RMSF values of the residues were estimated to be less than 0.3 nm, indicating a high level of stability in the protein backbone. This finding is consistent with the results obtained from the RMSD and Rg analyses. A slight elevation was seen in the residue LEU139 in comparison to the other residues, measuring around 0.49 nm with respect to the backbone and 0.57 nm with respect to the protein. Nevertheless, this residue is not present in close proximity to the binding pocket. The stability of Indolizine, 2-(4-methylphenyl), was assessed by examining the residues, namely those present in the active site. The distances between these residues were measured and found to be as follows: 0.102nm for Asp193, 0.0816nm for Ile189, 0.117nm for Lys59, 0.0829nm for Ser192, 0.0905nm for Val105, 0.0752nm for Leu182, 0.0672nm for Gly111, and 0.255nm for Leu131.

The CIE values for Indolizine, 2-(4-methylphenyl), have been seen to vary within the range of -84717.6 kJ/mol to -84887.4 kJ/mol, with an average value of -84322.4 kJ/mol. Additionally, the CIE plots for this compound exhibit a similar pattern to the plots of the calculated lowest LJIE for Indolizine, 2-(4-methylphenyl), showing a decrease in energy after 57000 ps. The lower limit of the LJIE for Indolizine, namely 2-(4-methylphenyl), has been seen to range from -8236.42 KJ/mol to -8662.04 KJ/mol, with an average value of -8493.2 KJ/mol. Based on the observed interaction energies, it is justifiable to infer that the ligand exhibits interaction with the protein.

The total binding free energies of the selected bioactive molecule were determined using the MMPBSA method which showed that RET – Indolizine, 2-(4-methylphenyl) complex depicts the total binding energy (BFE), Van der Waals energy, electrostatic energy, polar solvation energy contains -12.20, -25.35, -9.11 and -2.66 respectively represented in Table 12. The calculation of free binding energy (G_binding_) results indicates that the total binding free energy calculation involving the residues Asp193, Ile189, Lys59, Ser192, Val105, Leu182, Gly111, and Leu131 yields a value of -12.20 kcal/mol. This suggests a significant interaction between the protein RET and Indolizine, 2-(4-methylphenyl). Notably, the van der Waals interaction energy is found to be the highest, with a value of **-**25.35 kcal/mol, followed by the electrostatic energy with a value of -9.11 kcal/mol. These findings highlighted the strong interaction between the key protein residues and the ligand.

ADMET analysis shows strong drug-likeness and pharmacokinetic properties. It followed all five rule of Lipinski and no violation. According to pharmacokinetic analysis and compound interaction, it showed low glycoprotein permeability and CYP2C9 and CYP2C19 had no inhibitory effect. Furthermore, toxicity screening was performed through Protox-II tool and it depicted that there is low predictability against hepatoxicity, carcinogenicity, and immunotoxicity of Indolizine, 2-(4-methylphenyl)-. The mutagenicity and cytotoxicity score are also very low and inactive. Table 13 illustrate that there is a high indication that Indolizine, 2-(4-methylphenyl)- will not be carcinogenic, mutagenic, or hepatotoxic.

**Conclusion**

This study was used to analyze the bioactivity of *C. militaris.* The test includes the antioxidant activity using non-enzymatic assay, antimicrobial activity by using resistant and non-resistant strains. In biological activities, methanol extract of *C. militaris* shoes strong antioxidant and antibacterial activities as cordycepin and other phenolic compounds attributed many of the antioxidant properties to the compounds due to their strong hydrogen donating ability. Therefore, these findings suggest that *C. militaris* is an excellent candidate for the treatment of various ailments especially those for which antioxidant stress is one of the mechanisms for its occurrence. It was revealed that the indolizine, 2-(4-methylphenyl) has most binding affinity (micromolar) and optimized properties to be selected as the lead inhibitor. It interacts favorably with the active site of RET gene of NSCLC and is neuroprotective and hepatoprotective. So, the Indolizine, 2-(4-methylphenyl) is the potential inhibitor of RET gene to treat the N NSCLC. So, it was concluded that the pharmacological and medicinal potentials of *C. militaris* are auspicious as a versatile therapeutic mushroom due to the presence of bioactive compounds and it should be further investigated.

**Supporting information**

**S1 Table. GC-MS analysis of methanol extract of *C. militaris*.**

**S2 Table. LC-MS analysis of methanol extract of *C. militaris.***

**S3 Table. Binding pockets position and their scores of Aldose Reductase protein by DeepSite.**

**S4 Table. Molecular Docking of Bioactive Compound of GC-MS analysis with RET, PIK3CA and TCTN3 Gene.**

**S1 Figure. Chromatogram of GC-MS analysis.**

**S2 Figure. Antibacterial activity of methanolic extract of *C. militaris* against bacterial strains.** (A) *E. coli* (B) *K. pneumoniae* (C) *S. viridans* (D) *S. aureus*.

**Data availability statement**

More data can be accessed upon a reasonable request to the corresponding authors.

**Author contributions**

**Conceptualization:** Dr Muhammad Afzal; Kirnpal Kaur Banga Singh

**Formal analysis:** Neelam Shehzadi; Sarmir khan; Noshaba Mehmood

**Investigation:** Neelam Shehzadi; Yalnaz Naseem

**Project administration:** Muhammad Afzal; Naveed Ahmed

**Writing – original draft:** Neelam Shehzadi; Sarmir khan; Noshaba Mehmood; Yalnaz Naseem

**Writing – review & editing:** Mai Abdel Haleem A. Abusalah; Kirnpal Kaur Banga Singh; Naveed Ahmed

**Funding**

None.

**Acknowledgments**

The authors greatly acknowledge and express their gratitude to the Research work which was supported by University of Central Punjab, Lahore, Pakistan and collaboration with The First Affiliated Hospital of Zhengzhou University & Institute of Reproductive Health, Henan Academy of Innovations in Medical Science, NHC Key Laboratory of Birth Defects Prevention & Institute of drug discovery and development, Zhengzhou University, Zhengzhou 450001, China in computationally analysis.

**Conﬂict of interest**

The authors declare that they have no known competing financial interests or personal relationships that could have appeared to influence the work reported in this paper.

**References**

1.	Gohar D, Põldmaa K, Tedersoo L, Aslani F, Furneaux B, Henkel TW, et al. Global diversity and distribution of mushroom‐inhabiting bacteria. Environmental Microbiology Reports. 2022;14(2):254-64.

2.	Marie SM, Al Anbagi RA, Suliaman SQ. Silver Nanoparticles Synthesized from Polyporus plorans, A wild Mushroom with Detecting Its Physio-Chemical Characterization and Antimicrobial Activities. Egyptian Academic Journal of Biological Sciences, G Microbiology. 2023;15(1):43-59.

3.	Fokunang ET, Annih MG, Abongwa LE, Bih ME, Vanessa TM, Fomnboh DJ, et al. Medicinal mushroom of potential pharmaceutical toxic importance: contribution in phytotherapy. Functional Food. 2022;21(5):178-385.

4.	Park H-J. Current uses of mushrooms in cancer treatment and their anticancer mechanisms. International Journal of Molecular Sciences. 2022;23(18):105-302.

5.	Elkhateeb WA, Daba G. Cordyceps more than edible mushroom—a rich source of diverse bioactive metabolites with huge medicinal benefits. Journal of Biomedical Research and Environmental Sciences. 2022;3(5):566-74.

6.	Elkhateeb WA, Daba GM, Thomas PW, Wen T-C. Medicinal mushrooms as a new source of natural therapeutic bioactive compounds. Egyptian Pharmaceutical Journal. 2019;18(2):88-101.

7.	Holliday J. Cordyceps: a highly coveted medicinal mushroom. Medicinal Plants and Fungi: Recent Advances in Research and Development. 2017;36(4):59-91.

8.	Xu G, Du P, An L, Lv G, Miao L, Guangxin Y, editors. Research progress of active polypeptides of Cordyceps militaris. BIO Web of Conferences; 2017: EDP Sciences.

9.	Yang Q, Yin Y, Yu G, Jin Y, Ye X, Shrestha A, et al. A novel protein with anti-metastasis activity on 4T1 carcinoma from medicinal fungus Cordyceps militaris. International journal of biological macromolecules. 2015;80(8):385-91.

10.	Gong P, Wang S, Liu M, Chen F, Yang W, Chang X, et al. Extraction methods, chemical characterizations and biological activities of mushroom polysaccharides: A mini-review. Carbohydrate Research. 2020;494(9):108-637.

11.	Garić D, Dumut DC, Centorame A, Radzioch D. Western Blotting with Fast SDS‐PAGE and Semi‐Dry Protein Transfer. Current Protocols. 2023;3(7):833-990.

12.	Shamszadeh S, Akrami M, Asgary S. Size-dependent bioactivity of electrosprayed core–shell chitosan-alginate particles for protein delivery. Scientific Reports. 2022;12(1):200-397.

13.	Sies H, Belousov VV, Chandel NS, Davies MJ, Jones DP, Mann GE, et al. Defining roles of specific reactive oxygen species (ROS) in cell biology and physiology. Nature Reviews Molecular Cell Biology. 2022;23(7):499-515.

14.	Jia W, Wu X, Kang X. Integrated the embedding delivery system and targeted oxygen scavenger enhances free radical scavenging capacity. Food Chemistry: X. 2023;43(6):100-558.

15.	Chen C, Han Y, Li S, Wang R, Tao C. Nutritional, antioxidant, and quality characteristics of novel cookies enriched with mushroom (Cordyceps militaris) flour. CyTA-Journal of Food. 2021;19(1):137-45.

16.	Zhao H, Li M, Liu L, Li D, Zhao L, Wu Z, et al. Cordyceps militaris polysaccharide alleviates diabetic symptoms by regulating gut microbiota against TLR4/NF-κB pathway. International Journal of Biological Macromolecules. 2023;230(6):123-241.

17.	Kiely LJ, Hickey RM. Characterization and analysis of food-sourced carbohydrates. Glycosylation: Methods and Protocols. 2022;45(3):67-95.

18.	Rao AS, Nair A, Salu HA, Pooja K, Nandyal NA, Joshi VS, et al. Carbohydrases: a class of all-pervasive industrial biocatalysts. Biotechnology of Microbial Enzymes. 3: Elsevier; 2023. p. 497-523.

19.	Kashtoh H, Baek K-H. Recent updates on phytoconstituent alpha-glucosidase inhibitors: An approach towards the treatment of type two diabetes. Plants. 2022;11(20):27-220.

20.	Nölting S, März C, Jacob L, Persicke M, Schneiker-Bekel S, Kalinowski J. The 4-α-Glucanotransferase AcbQ Is Involved in Acarbose Modification in Actinoplanes sp. SE50/110. Microorganisms. 2023;11(4):848-987.

21.	Rabha J, Chetri BK, Das S, Jha DK. In-vitro and in-silico evaluation of antimicrobial and antibiofilm secondary metabolites of a novel fungal endophyte, Albophoma sp. BAPR5. South African Journal of Botany. 2023;158(4):347-68.

22.	Hasnain M, Jamsheed RA, Hussain Z, Latif R. A comparative study on Cordyceps militaris and Ophiocordyceps sinensis. International Journal of Natural Medicine and Health Sciences. 2023;2(2):1-6.

23.	Vo-Nguyen H-V, Nguyen B-HT, Do-Pham G-T, Luu TXT. Extracts from Cordyceps militaris: Novel, potential, and efficiently bioactive natural materials used for skin-care products. Microorganisms. 2023;24(4):342-532.

24.	Alizadeh Behbahani B, Rahmati-Joneidabad M, Noshad M. Ethanolic extract of Prosopis farcta root: Determination of total phenols and flavonoids, radical scavenging ability and its antimicrobial effect on some bacteria causing infection and food poisoning. Iranian Food Science and Technology Research Journal. 2023.

25.	Agarwal A, Gupta V, Yadav AN, Sain D, Rahi RK, Bera SP, et al. ASPECTS OF MUSHROOMS AND THEIR EXTRACTS AS NATURAL ANTIMICROBIAL AGENTS: Microbiology. Journal of microbiology, biotechnology and food sciences. 2023;12(6):9191-.

26.	Perumalsamy R, Krishnadhas L. Anti-diabetic activity of silver nanoparticles synthesized from the hydroethanolic extract of Myristica fragrans seeds. Applied Biochemistry and Biotechnology. 2022;194(3):1136-48.

27.	Chandrawanshi NK, Deepali AK, Dewhare SS, Verma S, Mahish PK, Kumar A. Bioactivity of essential oils–anticancer, anti-HIV, antiparasitic, anti-inflammatory, and other activities. Essential Oils: Sources, Production and Applications. 2023;9(3):133-489.

28.	Lee J, Lee H, Lee J, Chang P-S. Heterologous expression, purification, and characterization of a recombinant Cordyceps militaris lipase from Candida rugosa-like family in Pichia pastoris. Enzyme and Microbial Technology. 2023;168(4):110-254.

29.	Dos Santos AM, Albuini FM, Barros GC, Pereira OL, da Silveira WB, Fietto LG. Identification of the main proteins secreted by Kluyveromyces marxianus and their possible roles in antagonistic activity against fungi. FEMS Yeast Research. 2023;23(2):340-578.

30.	Mollahosseini A, Abdelrasoul A, Shoker A. Latest advances in zwitterionic structures modified dialysis membranes. Materials Today Chemistry. 2020;15:100227.

31.	Ajayan K, Sainath S, Sadik A, Nair MM, Nair AM, Vijayakumar A, et al. Bioconjugation of Meldrum's acid activated furan: A detergent compatible assay for protein quantitation. Analytical Biochemistry. 2023;662(2):114998.

32.	Dumut DC, Garić D, Centorame A, Radzioch D. The gradient-like separation and reduced running time with Tris-Tricine-HEPES buffer for SDS-PAGE. Analytical Biochemistry. 2022;653(6):114-789.

33.	Saravanan R, Raja K, Shanthi D. GC–MS Analysis, molecular docking and pharmacokinetic properties of phytocompounds from Solanum torvum unripe fruits and its effect on breast cancer target protein. Applied Biochemistry and Biotechnology. 2022;194(1):529-55.

34.	Al Azzam KM, Negim E-S, Aboul-Enein HY. ADME studies of TUG-770 (a GPR-40 inhibitor agonist) for the treatment of type 2 diabetes using SwissADME predictor: In silico study. Journal of Applied Pharmaceutical Science. 2022;12(4):159-69.

35.	Shobar SR, Naghdi S, Moghadam AT. Purification and characterization of pepsin enzyme from Scomberomorus commerson mackerel’s stomach by aluminum hydroxide, and improved its thermal stability by graphene oxide nanosheet. Journal of Ethnobiology and Ethnomedicine. 2023;12(7):321-432.

36.	Ballatore MB, del Rosario Bettiol M, Braber NLV, Aminahuel CA, Rossi YE, Petroselli G, et al. Antioxidant and cytoprotective effect of peptides produced by hydrolysis of whey protein concentrate with trypsin. Food Chemistry. 2020;319(10):126-472.

37.	Baidamshina DR, Koroleva VA, Olshannikova SS, Trizna EY, Bogachev MI, Artyukhov VG, et al. Biochemical properties and anti-biofilm activity of chitosan-immobilized papain. Marine Drugs. 2021;19(4):197-280.

38.	Seethapathy P, Pandita A, Pandita D, Sankaralingam S, Balasundaram H, Loganathan K. Cordyceps (Ophiocordyceps sinensis). Mushrooms: Nutraceuticals and Functional Foods. 2023;12(5):123-78.

39.	Ahmed SST, Fahim JR, Abdelmohsen UR. Chemical and biological studies on Allium sativum L.(1952-2020): A comprehensive review. Journal of advanced Biomedical and Pharmaceutical Sciences. 2022;5(1):1-22.

40.	Namdeo N, Arora R, Jha H, Namdeo N, Arora R. Biomedical Application of Advanced Microbial Approaches: Nutraceuticals, Biomedicine, and Vaccine Development. Industrial Microbiology and Biotechnology: Emerging concepts in Microbial Technology. 23: Springer; 2023. p. 273-97.

41.	Daba G. The endless nutritional and pharmaceutical benefits of the Himalayan gold, Cordyceps; Current knowledge and prospective potentials. Asian Journal of Natural Product Biochemistry. 2020;18(2):340-489.

42.	Tan L, Liu S, Li X, He J, He L, Li Y, et al. The large molecular weight polysaccharide from wild cordyceps and its antitumor activity on H22 tumor-bearing mice. Molecules. 2023;28(8):33-51.

43.	El-Ramady H, Abdalla N, Badgar K, Llanaj X, Törős G, Hajdú P, et al. Edible mushrooms for sustainable and healthy human food: nutritional and medicinal attributes. Sustainability. 2022;14(9):49-167.

44.	Pintathong P, Chomnunti P, Sangthong S, Jirarat A, Chaiwut P. The feasibility of utilizing cultured Cordyceps militaris residues in cosmetics: Biological activity assessment of their crude extracts. Journal of Fungi. 2021;7(11):973-1056.

45.	Fattah MA, El Baz M, Sherif A, Adel A. Complement components (C3, C4) as inflammatory markers in asthma. The Indian Journal of Pediatrics. 2010;77:771-3.

46.	Karimi F, Hamidian Y, Behrouzifar F, Mostafazadeh R, Ghorbani-HasanSaraei A, Alizadeh M, et al. An applicable method for extraction of whole seeds protein and its determination through Bradford's method. Food and Chemical Toxicology. 2022;164(4):113-453.

**Figure Legends:**

**Fig 1. FTIR analysis of methanol extract of *C. militaris.***

**Fig 2.** (A) BSA Standard Curve by Bradford assay. (B) RMSD stability study of Indolizine, 2-(4-methylphenyl). This manifest the RMSD in ‘nm’ of Indolizine, 2-(4-methylphenyl) following its alignment to the Time (ns). These findings determined the extent to which Indolizine, 2-(4-methylphenyl) binds to the protein (C) Detection of RMSF with protein backbone.

**Fig 3. SDS-PAGE Analysis.** (A) SDS-PAGE of *Cordyceps militaris* protein (B) Thermo Scientific ^TM^PageRuler^TM^ pre-stained Protein Ladder size (kDa) (C) Proteolytic enzyme treatment of Cordyceps protein.

**Fig 4. Molecular interactions of Indolizine, 2-(4-methylphenyl)- and RET.** (A) Binding pockets of RET protein by DeepSite. Active residues are highlighted by yellow arrows (B) H-bond showing 3-dimensional image of docked complex (C) 3D image of docked complex showing amino acids and distance of protein-ligand interaction (D) Visualization of best pose of Indolizine, 2-(4-methylphenyl) in complex with RET gene.

**Fig 5. Radius of gyration (Rg) and** **protein-ligand interaction energy (IE).** (A) Radius of gyration (Rg) for protein (B) Radius of gyration (Rg) for ligand (C) Coulombic interaction energy (CIE), (D) Lennard-Jones interaction energy (LJIE).

**Fig 6. SwissADME analysis.** (A) Boiled egg analysis of Indolizine, 2-(4-methylphenyl)- (B) Swiss Target Prediction.
