# Supplementary material for: Investigation of biometabolites and novel antimicrobial peptides derived from promising source Cordyceps militaris and effect of non-small cell lung cancer genes computationally
Source: PLoS One. 2025 Jan 23;20(1):e0310103. doi: 10.1371/journal.pone.0310103 (PMC11756765; doi:10.1371/journal.pone.0310103)
Supplement: Attachment — Submitted filename: Response to Reviewer1.docx [file pone.0310103.s009.docx]

Journal Requirements:

Please review your reference list to ensure that it is complete and correct. If you have cited papers that have been retracted, please include the rationale for doing so in the manuscript text, or remove these references and replace them with relevant current references. Any changes to the reference list should be mentioned in the rebuttal letter that accompanies your revised manuscript. If you need to cite a retracted article, indicate the article’s retracted status in the References list and also include a citation and full reference for the retraction notice.

Author response: Dear editorial members, thank you so much for allowing us to make the manuscript better for the reader and scientific community. We have revised the manuscript as per the comments from the editor and reviewers and we have attached a rebuttal letter in the submission portal. We have highlighted all of the changes in red colour and in track change format. Furthermore, we have counterchecked the cited references and we did not noticed any retraction for them.

**Additional Editor Comments:**

The authors have revised the manuscript according to the comments provided by the reviewers. However, it still needs minor revisions as per the additional reviewers comments and my concerns. I recommend to enrich the introduction part by including the following contents and references.

Author response: Dear editor, thank you so much for allowing us to make the manuscript better for the reader and scientific community. We have revised the manuscript as per the comments from the editor and reviewers and we have attached a rebuttal letter in the submission portal. We have highlighted all of the changes in red colour and in track change format.

1. Genomics and metabolomics have been extensively investigated for the discovery of new drugs particularly in the discovery of peptide-based drugs. Add few lines about the same and cite the below references

a. Jiang, M., Chen, S., Lu, X., Guo, H., Chen, S., Yin, X.,... Liu, L. (2023). Integrating Genomics and Metabolomics for the Targeted Discovery of New Cyclopeptides with Antifungal Activity from a Marine-Derived Fungus Beauveria felina. Journal of Agricultural and Food Chemistry, 71(25), 9782-9795. doi: 10.1021/acs.jafc.3c02415.

b. Kloosterman, A.M., Medema, M.H. and van Wezel, G.P., 2021. Omics-based strategies to discover novel classes of RiPP natural products. Current Opinion in Biotechnology, 69, pp.60-67.

c. Xu, T., Yan, X., Kang, A., Yang, L., Li, X., Tian, Y.,... Guo, Y. (2024). Development of Membrane-Targeting Fluorescent 2-Phenyl-1H-phenanthro[9,10-d]imidazole-Antimicrobial Peptide Mimic Conjugates against Methicillin-Resistant Staphylococcus aureus. Journal of Medicinal Chemistry, 67(11), 9302-9317. doi: <https://doi.org/10.1021/acs.jmedchem.4c00436>

d. He, B., Lang, J., Wang, B., Liu, X., Lu, Q., He, J.,... Yang, J. (2020). TOOme: A Novel Computational Framework to Infer Cancer Tissue-of-Origin by Integrating Both Gene Mutation and Expression. Frontiers in Bioengineering and Biotechnology, 8. doi: 10.3389/fbioe.2020.00394

e. Ali, A.M., Atmaj, J., Van Oosterwijk, N., Groves, M.R. and Dömling, A., 2019. Stapled peptides inhibitors: a new window for target drug discovery. Computational and structural biotechnology journal, 17, pp.263-281.

Author response: Dear editor, thank you for your valuable suggestion to improve the writeup by providing some background about the genomics and metabolomics. We have amended the discussion section and have cited the relevant new references to strengthen the statements. (Line 560-568)

2. Some of the important works related to the therapeutic modalities in non-small cell lung cancer were not referred. These must be discussed and cited in the introduction.

a. Zhao, Y., Chen, S., Shen, F., Long, D., Yu, T., Wu, M.,... Lin, X. (2019). In vitro neutralization of autocrine IL‑10 affects Op18/stathmin signaling in non‑small cell lung cancer cells. Oncol Rep, 41(1), 501-511. doi: 10.3892/or.2018.6795

b. Chen, S., Zhao, Y., Shen, F., Long, D., Yu, T.,... Lin, X. (2019). Introduction of exogenous wild‑type p53 mediates the regulation of oncoprotein 18/stathmin signaling via nuclear factor‑κB in non‑small cell lung cancer NCI‑H1299 cells. Oncol Rep, 41(3), 2051-2059. doi: 10.3892/or.2019.6964

c. Dai, J., Ashrafizadeh, M., Aref, A. R., Sethi, G., & Ertas, Y. N. (2024). Peptide-functionalized, -assembled and -loaded nanoparticles in cancer therapy. Drug Discovery Today, 29(7), 103981. doi: <https://doi.org/10.1016/j.drudis.2024.103981>

d. Tan, T., Feng, Y., Wang, W., Wang, R., Yin, L., Zeng, Y.,... Xie, T. (2023). Cabazitaxel-loaded human serum albumin nanoparticles combined with TGFβ-1 siRNA lipid nanoparticles for the treatment of paclitaxel-resistant non-small cell lung cancer. Cancer Nanotechnology, 14(1), 70. doi: <https://doi.org/10.1186/s12645-023-00194-7>

e. Lou, J., Zhao, L., Huang, Z., Chen, X., Xu, J., TAI, W. C.,... Xie, T. (2021). Ginkgetin derived from Ginkgo biloba leaves enhances the therapeutic effect of cisplatin via ferroptosis-mediated disruption of the Nrf2/HO-1 axis in EGFR wild-type non-small-cell lung cancer. Phytomedicine, 80, 153370. doi: <https://doi.org/10.1016/j.phymed.2020.153370>

Author response: Dear editor, thank you for your valuable suggestion to improve the discussion section. We have amended the discussion section and have cited the relevant new references to strengthen the statements. A new paragraph has been added on the impact of Cordyceps militaris of Non-Small Cell Lung Cancer Genes. (Line 100-110)

Reviewer #3:

The topic to which the manuscript “Investigation of Biometabolites and Novel Antimicrobial Peptides Derived from Promising Source Cordyceps militaris and Effect of Non-Small Cell Lung Cancer Genes Computationally” is devoted is relevant and of practical importance. Due to a wide range of bioactive compounds, C. militaris has significant potential for pharmacological use. The manuscript is of a modern scientific level, impresses with its instrumental level of execution and depth of research.This work definitely deserves to be published and the results highlighted. However, there are a number of recommendations that would improve the introduction.

Author response: Dear reviewer, thank you so much for valuable words, to review our manuscript, and to provide us with your valuable comments. The manuscript became better for the reader and scientific community. We have revised the manuscript as per the comments from the you and other reviewers and we have attached a rebuttal letter in the submission portal. We have highlighted all of the changes in red colour and in track change format.

The article is devoted to the study of the impact of Cordyceps militaris of Non-Small Cell Lung Cancer Genes. However, the introduction does not contain any information about the already published studies of the effect of this mushroom on non-small cell lung cancer. This information should be added and I recommend pay attention to the following works:

Luo L, Ran R, Yao J, Zhang F, Xing M, Jin M, Wang L, Zhang T. Se-Enriched Cordyceps militaris Inhibits Cell Proliferation, Induces Cell Apoptosis, And Causes G2/M Phase Arrest In Human Non-Small Cell Lung Cancer Cells. Onco Targets Ther. 2019;12:8751-8763 <https://doi.org/10.2147/OTT.S217017>

Jo E, Jang HJ, Shen L, Yang KE, Jang MS, Huh YH, Yoo HS, Park J, Jang IS, Park SJ. Cordyceps militaris Exerts Anticancer Effect on Non-Small Cell Lung Cancer by Inhibiting Hedgehog Signaling via Suppression of TCTN3. Integr Cancer Ther. 2020 Jan-Dec;19:1534735420923756. doi: 10.1177/1534735420923756.

Lu YY, Huang X, Luo ZC, et al. [Mechanism of Cordyceps militaris against non-small cell lung cancer: based on serum metabolomics]. Zhongguo Zhong yao za zhi = Zhongguo Zhongyao Zazhi = China Journal of Chinese Materia Medica. 2022 Sep;47(18):5032-5039. DOI: 10.19540/j.cnki.cjcmm.20220613.702.

Author response: Dear reviewer, thank you for your valuable suggestion to improve the introduction section. We have amended the introduction section and have cited the relevant new references to strengthen the statements. Line 100-110.

In my opinion, the information about the SDS-PAGE method (line 75-79) is unnecessary in the introduction. It is logical to provide this information in the discussion.

Author response: Dear reviewer, the sentence has been removed from the revised version of manuscript.

Line 112-113: The mushroom was taxonomically classified, identified and 113 confirmed from the University of Central Punjab, Lahore Pakistan.

Was the sample genetically identified? Was the data entered into the genebank? If yes, please provide the sample number.

Author response: Dear reviewer, in the current study, the plant sample was not genetically identified. Hence, we don’t have any data to be deposited on genbank database. The plant sample was identified by a botanist at University of Central Punjab and was given a voucher number for future references. All of the other experiments were conducted only after the confirmation from the botanist.
